# Effect of GLP-1 receptor agonists on weight and cardiovascular outcomes: A review

**DOI:** 10.1097/MD.0000000000040364

**Published:** 2024-11-01

**Authors:** Fatima Ali Raza, Rafiya Altaf, Talha Bashir, Fatima Asghar, Rabiya Altaf, Sohaib Tousif, Aman Goyal, Aisha Mohammed, Mahnoor Faisal Mohammad, Mahfuza Anan, Sajjad Ali

**Affiliations:** a Department of Medicine, Karachi Medical and Dental College, Karachi, Pakistan; b Department of Surgery, Dow International Medical College, Dow University of Health Sciences, Karachi, Pakistan; c Department of Medicine, Karachi Institute of Medical Sciences, Combined Military Hospital Malir, Karachi City, Pakistan; d Department of Medicine, Ras Al Khaimah College of Medical Sciences, Ras Al Khaimah Medical and Health Sciences University, Ras Al Khaimah, United Arab Emirates; e Department of Medicine, Mersey and West Lancashire Teaching Hospitals NHS Trust, Prescot, United Kingdom; f Department of Medicine, Ziauddin University, Karachi City, Pakistan; g Department of Medicine, Seth Gordhandas Sunderdas Medical College and King Edward Memorial Hospital, Mumbai, India; h Department of Medicine, Comanche County Memorial Hospital, Lawton, OK; i Department of Medicine, Liaquat National Hospital and Medical College, Karachi, Pakistan; j Department of Medicine, Bangladesh Medical College, Dhaka, Bangladesh; k Department of Medicine, Ziauddin University, Karachi City, Pakistan.

**Keywords:** cardiovascular, GLP-1 agonists, outcomes, weight

## Abstract

Diet and lifestyle modifications remain the foundation of obesity treatment, but they have historically proven insufficient for significant, long-term weight loss. As a result, there is a high demand for new pharmacologic treatments to promote weight loss and prevent life-threatening diseases associated with obesity. Researchers are particularly interested in 1 type of drug, glucagon-like peptide 1 receptor agonists (GLP-1 RAs), because of its promising potential in addressing the limitations of non-pharmacologic treatments. In addition to their role in weight loss, these drugs have shown promising early evidence of cardiovascular benefits in obese patients, further enhancing their clinical relevance. Semaglutide and liraglutide, which were initially approved for the treatment of type 2 diabetes, have since been approved by the Food and Drug Administration as weight loss medications due to their effectiveness in promoting significant and sustained weight loss. In this narrative review, we will explore the mechanism of GLP-1 RAs, their effects on weight loss, cardiovascular risk factors and outcomes, common adverse effects, and strategies for managing these effects.

## 1. Introduction

Obesity is a major risk factor for cardiovascular disease (CVD) and heart failure, which contributes to higher morbidity and mortality rates worldwide.^[1]^ Historically, weight management strategies have included a variety of approaches, including bariatric surgery and pharmacological treatments; however, these methods have frequently encountered practical limitations or side effects, making them less effective or acceptable.^[2]^ Recent advances in pharmacotherapy have introduced GLP-1 receptor agonists (GLP-1 RAs) and gastric inhibitory polypeptide (GIP) receptor agonists, which have shown promise in both weight loss and CVD prevention.^[3]^ These agents work by increasing insulin secretion, decreasing appetite, and improving glucose metabolism, with a growing number of GLP-1 RAs and GIP analogs now on the market, including liraglutide, semaglutide, and tirzepatide.^[4]^ Ongoing and future trials are investigating their long-term efficacy and safety profiles, with a focus on cost, patient adherence, and side effects.^[5]^ This review investigates the mechanism of glucagon-like peptide 1 receptor agonist (GLP-1 RA), its effects on weight loss, cardiovascular risk (CV) factors and outcomes, common adverse effects and their management, and the drugs’ benefits and limitations, emphasizing the importance of careful patient selection.

## 2. The link between obesity, CVD, and diabetes

A large body of epidemiological and clinical evidence suggests a link between obesity and CVDs, including hypertension, stroke, coronary heart disease, and, most importantly, heart failure.^[6]^

### 2.1. Epidemiological evidence

In a meta-analysis comparing new-onset HF among normal-weight and overweight patients, being overweight increased the risk of developing HF by 33%.^[7]^ According to the Framingham Heart Study, each 1-unit increase in body mass index (BMI) is associated with a 5% increased HF risk in men and 7% in women, indicating a dose-dependent relationship.^[8]^ Statistics show that each 5 kg/m^2^ increase in BMI increases the risk of hemorrhagic stroke and coronary heart disease by 10% and 15%, respectively.^[9]^

### 2.2. Insulin resistance and CV

Another major concern with weight gain is its direct linear relationship with systolic and diastolic blood pressure (BP), which contributes to a significantly increased risk of primary hypertension.^[10]^ As a result, excess weight gain accounts for 78% of primary (essential) hypertension cases in men and 65% in women.^[11]^ Furthermore, a meta-analysis conducted by Zhu et al^[12]^ found a strong correlation between the prevalence of myocardial infarction (MI) and obesity, as well as the importance of maintaining weight as a preventative measure. Abdominal obesity increases the risk of insulin resistance, a metabolic syndrome component that precedes the development of type 2 diabetes (T2D).^[13]^ Insulin resistance also increases the likelihood of other metabolic risk factors such as atherogenic dyslipidemia, hypertension, glucose intolerance, and a prothrombotic state, all of which are indirect risk factors for CVD.^[14]^ In essence, patients with abdominal obesity are more likely to develop T2D,^[15]^ which puts them at a higher risk of adverse CV events than normoglycemic subjects.^[16]^ The San Antonio Heart Study adds to this notion by demonstrating a 2- to 3-fold increase in mortality risk in subjects with diabetes or metabolic syndrome at baseline.^[17]^

### 2.3. The impact of weight management on CVD

The question of whether obesity’s associated factors increase the risk of CVD or if obesity is the sole culprit is a source of ongoing debate. Some argue that epidemiological studies are skewed and that a phenotype known as “metabolically healthy obesity” exists, implying that the subset of obese people who do not have any other cardiometabolic abnormalities (such as hypertension, CVD, T2D, or hyperlipidemia) may not be at an increased risk of CVD or death. According to Mendelian randomization studies, the theory of “metabolically healthy obesity” has been debunked by demonstrating that the effect of obesity on CVD is causal.^[18]^ As a result, combating obesity is critical regardless of whether patients have CV risk factors or not, because significant weight loss may potentially reverse the hemodynamic changes that predispose to changes in cardiac performance and morphology.^[19]^

## 3. The need for weight loss and antidiabetic pharmacotherapy that improves cardiovascular outcomes

Because obesity is also associated with poor CV outcomes, it is a common misconception that simple weight loss interventions are the definitive solution to reversing these side effects. Even though diet and lifestyle changes remain the cornerstones of obesity treatment,^[20]^ they have historically proven to be insufficient for achieving significant long-term weight loss.^[21]^ Additional evidence calls into question the precise relevance of weight loss to CV outcomes overall. For example, the Look Action for Health in Diabetes trial, which was conducted to investigate the effects of lifestyle interventions on the rates of heart disease, stroke, and CV-related deaths in obese patients, found that weight loss had little to no effect on CV events, rendering it obsolete.^[22]^ Patients who lose a significant amount of weight (>10 kg) may have better CV outcomes, but this is only true for a small subset of the population. Thus, the addition of pharmacotherapy may result in greater and more sustained weight loss.

### 3.1. Early pharmacological interventions

As an alternative, drugs were studied in 2 categories: those that could potentially increase metabolism or those that could suppress appetite. The first drugs to aid in weight loss, such as thyroid hormone, amphetamines (which also suppress appetite), and dinitrophenol, had short-lived success due to the CV effects of thyroid hormones, the potential for abuse with amphetamines, neuropathy, and cataracts with dinitrophenol.^[23]^

### 3.2. Appetite suppressants

Unfortunately, the early appetite suppressants did not produce positive results either. In 1997, a widely used anti-obesity drug called fenfluramine, which was used in the combination drug fen-phen (fenfluramine and phentermine), was recalled in the United States after echocardiography revealed that 30% of patients had valvular heart disease.^[24]^ Sibutramine, a serotonin and epinephrine uptake inhibitor, was approved the same year for appetite suppression; however, it was discontinued in 2010 after a post-marketing study revealed statistical evidence of increased stroke and MIs in patients with preexisting CVD or diabetes.^[25]^ Lorcaserin was one of the most recent drugs to be removed from the market due to its questionable safety profile, which revealed an increased risk of cancer that far outweighed its appetite-suppressing benefit.^[26]^

### 3.3. Bariatric surgery

Bariatric Surgery, which encompasses restrictive procedures (adjustable peri-gastric ring and sleeve gastrectomy) and malabsorptive procedures (gastric bypass and biliary pancreatic shunts), is a more effective and long-term solution for patients in need of excessive weight loss.^[27]^ However, these surgeries come with a long list of postoperative risks, including bleeding, herniation, anastomotic leak, and “dumping syndrome,” to name a few.^[28,29]^ Furthermore, surgery is not considered the first line of treatment for everyone because it requires patients to meet the eligibility criteria, which include a BMI ≥40, the presence of one or more obesity-related comorbidities (heart disease, stroke, hypertension, and T2D), and the inability to maintain a healthy weight over time.^[30]^

### 3.4. Regulatory changes and safety trials

As previously discussed, CVD is linked to diabetes; in fact, CVD is the leading cause of death in patients with diabetes mellitus, emphasizing the importance of antidiabetic pharmacotherapy that not only maintains CV neutrality but also reduces CV risk.^[16]^ Until recently, antidiabetic drugs were not required to demonstrate CV safety. However, following the safety concerns raised by rosiglitazone and its potential side effects such as an increased risk of death and MI,^[31]^ the Food and Drug Administration (FDA) mandated that innovative antidiabetic therapies for T2D be scrutinized by conducting safety trials to confirm their CV neutrality.^[32]^ This is critical because certain antidiabetic medications can worsen heart failure or increase the risk of potentially fatal lactic acidosis.^[33]^

## 4. The interest in GLP-1 RAs as weight loss agents

Over the last decade, there have been several advances in the development of safe antidiabetic pharmacotherapy, resulting in the introduction of a class of incretin mimetic drugs; recently, 1 such specific drug, GLP-1 RA, has received significant attention due to its promising potential as a weight loss agent. Despite the fact that the GIP hormone was the first insulin-stimulating factor discovered in the 1960s,^[34]^ it demonstrated an impaired insulinotropic effect in patients with type 2 diabetes mellitus (T2DM), disappointing incretin enthusiasts.^[35]^

### 4.1. Discovery of glucagon-like peptide

Subsequent resection experiments revealed the existence of an additional incretin, glucagon-like peptide (GLP), which was an unexpected processing fragment of the newly discovered proglucagon.^[36]^ It was then isolated from the gut and found to stimulate insulin and inhibit glucagon secretion.^[37,38]^ Unlike GIP, this peptide could maintain its effects in patients with T2D and was quickly recognized as having remarkable antidiabetic effects in clinical studies.^[39]^ One major limitation in the use of GLP-1 peptide was its extremely short half-life (1.5–5 minutes) due to rapid cleavage and degradation by the enzyme dipeptidyl peptidase-4 (DPP-4),^[40]^ so DPP-4 resistant GLP-1 RAs were developed for longer-lasting effects.^[41]^

### 4.2. GLP-1 RAs in the treatment of T2DM and weight loss effects

GLP-1 RAs were licensed for the treatment of T2DM due to their ability to lower A1C and achieve the hemoglobin A1c (HbA1c) goal of <7%. They also normalize fasting and prandial blood glucose levels.^[42,43]^ It is suggested that starting GLP-1 RA with insulin as an add-on therapy before or after 90 days resulted in more patients achieving blood sugar levels of <7% or 8%, and patients lowering their blood sugar levels by ≤%1 to 2% at 6 and 12 months.^[44]^ Unexpectedly, trials on diabetic patients revealed impressive weight loss,^[45]^ sparking researchers’ interest in the potential use of GLP-1 RA drugs as weight loss therapy.

## 5. Pharmacodynamics of GLP-1 RA mediated weight loss

### 5.1. Mechanism of action

To understand their mechanism, we must first understand the normal physiology of the incretin molecule’s pathway, as well as the changes that GLP-1 RA undergoes during obesity. GLP-1 is produced by proglucagon cleavage in L-cells in the intestinal mucosa, α-cells in pancreatic islets, and neurons in the solitary tract nucleus.^[46]^ This hormone regulates energy homeostasis and feeding behavior by stimulating receptors on islet β-cells. It enhances insulin secretion, promotes β-cell proliferation, inhibits glucagon production, and increases resistance to apoptosis.^[47]^ In addition to its incretin effects, GLP-1 functions as a satiety signaling molecule, delaying gastric emptying and gastric acid secretion.^[48–50]^

### 5.2. Impact of obesity on GLP-1 function

**Figure 1.**
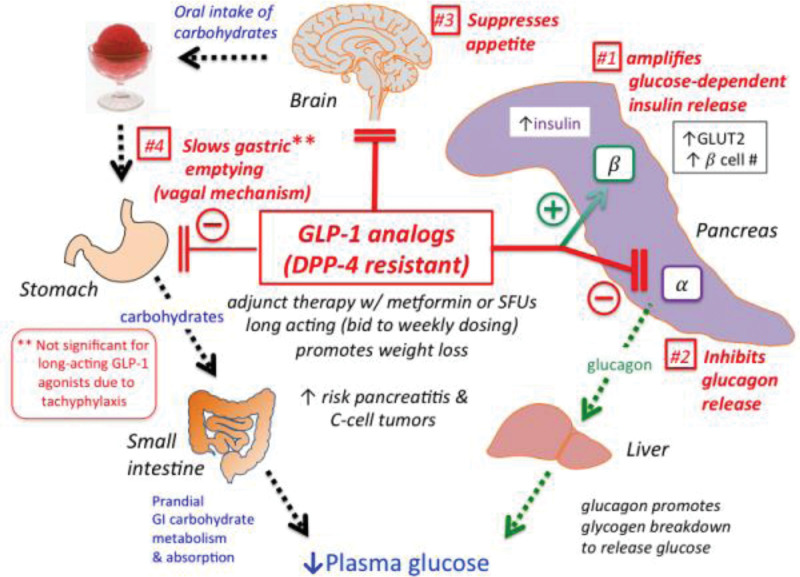
The mechanism of action of GLP-1 analogs (DPP-4 resistant). DPP-4 = dipeptidyl peptidase-4, GLP-1 = glucagon-like peptide.

Obesity, according to documented evidence, may contribute to GLP-1 function impairment. In a study of 51 subjects with BMIs ranging from 20 to 61 kg/m^2^, Muscelli et al discovered an inverse correlation between the incretin effect and BMI.^[51]^ The underlying pathophysiology for this reduced effect has not been definitively determined, but there are 2 plausible theories. The first is reduced GLP-1 secretion as a result of L-cells losing sensitivity to carbohydrates in obese people.^[52]^ Another reason for lower GLP-1 levels could be increased resistance to leptin, which is thought to regulate GLP-1 secretion.^[53]^ The second explanation for the decreased incretin effect in obese patients is a reduction in GLP-1’s insulinotropic effectiveness. This hypothesis is supported by the Knop et al study, which found that a decrease in the incretin effect was not accompanied by a decrease in the secretion of these hormones.^[54,55]^ Even though the exact underlying cause of impairment is unknown, it is undeniable that obesity influences GLP-1, as evidence shows that weight loss, whether diet-induced^[56]^ or following Roux-en-Y gastric bypass, can increase GLP-1 secretion.^[57]^ Finally, the impairment of GLP-1 and its effects on satiety explains why weight loss may appear impossible for obese people, trapping them in a never-ending cycle of overeating and weight gain that leads to additional incretin dysfunction. As a result, GLP-1 receptor analogs are required to aid in weight loss by pharmacologically reproducing appetite-suppressing effects such as delayed gastric emptying^[58]^ and direct stimulation of satiety centers in the hindbrain and hypothalamus^[59]^ (Fig. 1).

## 6. Cardiovascular effects of GLP-1 RAs

GLP-1 RA’s weight loss effect is thus well established in both diabetic and nondiabetic individuals. However, as previously discussed, weight loss without improvement in CV outcomes is futile. Once again, clinical applications of GLP-1 RA have demonstrated its status as the gold standard; despite being initially studied for incretin and weight loss effects, evidence has shown that GLP-1 RA is a multipurpose drug due to its non-glycemic effects, which can interact with CV risk factors while also providing beneficial effects on CV outcomes.^[60]^

### 6.1. Mechanisms of cardiovascular benefit

**Figure 2.**
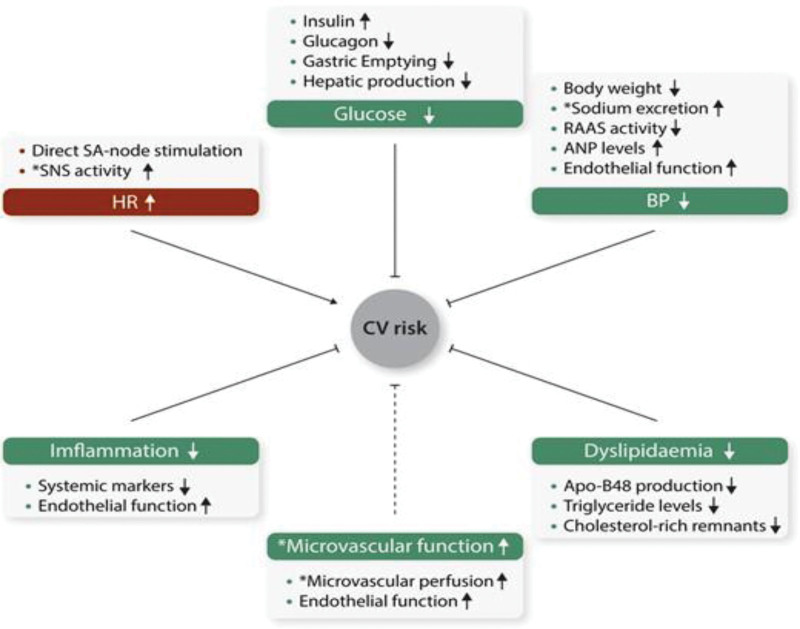
The direct and indirect effect of GLP-1R on the heart and vessels. GLP-1R = GLP-1 receptor.

The discovery of GLP-1R on cardiomyocytes, endothelial cells, and the autonomic nervous system strongly suggests direct and indirect effects on the heart and vessels (Fig. 2).^[61–64]^ To begin, GLP-1 improves microvascular blood flow by increasing endothelial function and microvascular perfusion.^[65–68]^ Long-term studies of GLP-1 RA showed moderate BP reductions, which could be attributed to altered salt homeostasis, weight loss, sympathetic nervous system involvement, or serum atrial natriuretic peptide.^[62]^

### 6.2. Impact on cardiovascular markers

The meta-analysis concludes that GLP-1 RAs exenatide and liraglutide reduced systolic and diastolic BP by 1 to 5 mm Hg in T2DM patients compared to other antidiabetic drugs.^[69]^ Another CV marker affected by the GLP-1 peptide is heart rate, which is acutely increased in response to a suggested increase in sympathetic nervous system activity or direct sinoatrial node stimulation.^[64,70,71]^ Finally, GLP-1 RA improves lipid profiles by lowering postprandial triglyceride levels, apo B-48, apo C-III, remnant lipoprotein cholesterol, and remnant lipoprotein triglycerides.^[72]^ Overall, placebo-controlled clinical trials with GLP-1 RA revealed a consistent 0.2–0.3 mmol/L decrease in triglyceride concentrations.^[73]^ Semaglutide treatment has been shown to significantly reduce postprandial triglyceride, very low-density lipoprotein, and apoB-48 levels.^[74]^ These findings clearly indicate that the potential anti-atherosclerotic effects of GLP-1 RA correspond to a decrease in lipids. As previously stated, GLP-1 RAs are clearly recognized for reducing weight and HbA1c, which may have a positive impact on CV outcomes overall.^[75,76]^

## 7. Currently approved GLP-1 RAs and their indications

Several different GLP-1 RA drugs have been developed and released into the market over the years. They are distinguished by their molecular structure, pharmacokinetics, administration, storage, efficacy, tolerability, patient satisfaction, half-life, similarity to GLP-1, clearance, dose, and short- or long-acting mechanisms.^[77]^

### 7.1. Overview of approved GLP-1 RAs

Exenatide (twice daily), liraglutide, lixisenatide (both once daily), oral semaglutide (once daily), and the once-weekly agents exenatide (Bydureon), albiglutide, semaglutide, and dulaglutide are among the GLP-1 RAs currently approved in the United States.^[78–85]^ These drugs were initially only used to treat T2D because they effectively reduced A1C and weight. The first GLP-1 RA drug to receive FDA approval and be officially classified as a weight loss medication was liraglutide.^[86]^

### 7.2. Liraglutide and semaglutide

In 2014, this under the skin injection was approved as an adjunct to diet and exercise for adults with a BMI of ≥30 (obese) or ≥27 (overweight) and at least one weight-related condition (hypertension, T2D, dyslipidemia). The initial dosage of liraglutide is 0.6 mg daily, regardless of meals, and is increased weekly by 0.6 mg until the target dosage of 3 mg per day is met. Patients who can tolerate the 3 mg dosage can continue with treatment, while others are advised to discontinue, as are patients who are unable to lose 4% of their baseline body weight after 4 months of treatment or who do not show weight fluctuations after a year of medication. Patients may experience gastrointestinal (GI) side effects including nausea, diarrhea, constipation, and vomiting. Furthermore, approximately 1 in every 4 patients with T2D will experience symptomatic hypoglycemia at some point during the year.^[87]^ Nonetheless, liraglutide remains the superior weight loss agent when compared to orlistat (Xenical).^[88]^ The next drug to join liraglutide on the list of GLP-1 RA drugs used for chronic weight management was weekly semaglutide, 2.4 mg.^[89]^ This agent is also injected subcutaneously (SC), meets the same inclusion criteria as liraglutide, and has nearly identical side effects, with nausea being the most common.^[90]^ In comparison to liraglutide, semaglutide causes significantly more weight loss but may also cause more side effects.^[91]^ semaglutide is the only GLP-1 RA that provides the advantages of a highly effective GLP-1 RA in both injectable and oral formulations^[92]^; however, oral semaglutide has yet to be approved as a weight loss medication.

### 7.3. Cardiovascular indications and future developments

Lastly, dulaglutide, liraglutide, and injectable semaglutide have been approved to reduce major adverse cardiovascular events (MACE) in adults with T2D who have established CVD or multiple CV risk factors.^[93–95]^ In contrast, none of the GIP RA drugs have been approved by the FDA, but tirzepatide, a dual-GIP and GLP-1 RA, is thought to have undiscovered potential. It is currently in phase 3 development for managing blood glucose in adults with T2D, chronic weight management, and heart failure with preserved ejection fraction (HFpEF).^[96]^

## 8. Trials involving GLP-1 RA

### 8.1. Semaglutide

#### 8.1.1. Cardiovascular outcome trials in patients with diabetes

The Trial to Evaluate Cardiovascular and Other Long-term Outcomes with semaglutide in Subjects with Type 2 Diabetes was a noninferiority trial that compared once-weekly injectable semaglutide (0.5 or 1.0 mg) to placebo in terms of CV safety over a 2.1-year median observation period.^[97]^ This trial included 3927 people with T2D, with 83% having preexisting CVD, chronic kidney disease, or both. Despite the fact that the goal of this study was not to determine superiority, the findings revealed a significant 26% reduction in the risk of the 3-point MACE endpoint among patients taking semaglutide. The reduction was primarily driven by a 39% decrease in nonfatal stroke (*P* = .04) and a 26% decrease in nonfatal MI (*P* = .12), with no significant effect on CV mortality (*P* = .79). Compared to placebo, semaglutide 0.5 and 1.0 mg significantly reduced body weight (−2.9 and −4.3 kg, respectively; *P* < .001) and A1C levels (−0.7% and −1.0%, respectively; *P* < .001 for both). Semaglutide 1.0 mg reduced mean systolic BP by 2.6 mm Hg compared to placebo (*P* < .001).

Similarly, Peptide Innovation for Early Diabetes Treatment 6 was a preapproved noninferiority trial with an identical endpoint to assess the CV safety of oral semaglutide versus placebo among patients with T2D who were at high CV risk.^[98]^ A total of 3183 patients (84.7% with established CVD or renal disease) were followed up for a median of 15.9 months. The 3-point MACE endpoint occurred in 3.8% of patients receiving oral semaglutide compared to 4.8% receiving placebo, demonstrating noninferiority (*P* < .001) but not superiority (*P* = .17). Analyzing the individual components of the primary outcome revealed a nominally significant reduction in the risk of dying from CV causes in the semaglutide group. At 62 weeks, the oral semaglutide group had lower glycated hemoglobin levels than the placebo group (−1.0% vs −0.3%), as well as lower body weight (−4.2 vs −0.8 kg). The oral semaglutide group had slightly lower systolic BP, low-density lipoprotein cholesterol, and triglyceride levels.

#### 8.1.2. Weight loss trials in patients with diabetes

The Semaglutide Treatment Effect in People with Obesity (STEP) 2 trial compared the efficacy and safety of once-weekly subcutaneous semaglutide 2.4 mg to semaglutide 1.0 mg and placebo for weight management in obese or overweight adults with T2D^[99]^ participants were considered eligible if they reported at least 1 unsuccessful dietary attempt to lose weight, had a body mass index of at least 27 kg/m^2^, an HbA1c of 7% to 10%, and had been diagnosed with T2D at least 180 days prior to screening. A total of 1210 participants were randomly assigned in a 1:1:1 ratio to receive either semaglutide 2.4 mg, semaglutide 1.0 mg, or a matching placebo once a week for 68 weeks, along with a lifestyle intervention that included dietary counseling and increased physical activity for 150 minutes weekly. The co-primary endpoints were percentage change in bodyweight and a weight loss of at least 5%. At week 68, the average change in body weight from baseline was −9.64% in the 2.4 mg semaglutide group and −3.42% in the placebo group (*P* < .001). Weight loss of ≥5% was more common in the 2.4 mg semaglutide group compared to the placebo group (68.8% vs 28.5%; *P* < .001). In conclusion, semaglutide 2.4 mg achieved a clinically significant decrease in bodyweight compared to placebo. At week 68, semaglutide 2.4 mg reduced HbA1c from baseline by −1.6%, resulting in more than two-thirds of patients reaching their target HbA1c of 6.5% or lower. Semaglutide 2.4 mg improved cardiometabolic risk factors, lipid profile, and inflammatory markers compared to placebo. Semaglutide 2.4 mg significantly improved systolic BP compared to placebo (−3.9 vs −0.5 mm Hg).

#### 8.1.3. Weight loss trials in patients with obesity

Similar to STEP 2, trials such as STEP 1, 3, 4, and 5 evaluate the efficacy of semaglutide at a higher dose of 2.4 mg/wk, specifically for promoting weight loss in obese patients who do not have diabetes.^[100–103]^ These phase 3 multicenter, randomized, double-blind trials are some of the most important clinical trial programs for obesity management. These trials have similar inclusion criteria: adults with at least 1 unsuccessful dietary effort, a BMI of ≥30 or ≥27 kg/m^2^, and 1 or more weight-related coexisting conditions (hypertension, CVD, or sleep apnea). These trials were only for people without diabetes, as those with a hemoglobin A1C of ≥6.5% were excluded. The primary endpoints for all of these trials were the percentage change in body weight from baseline to the end of the trial, as well as a weight loss of at least 5%, with the exception of the STEP 4 trial, which had only 1 endpoint: the percentage change in body weight from weeks 20 to 68. The STEP 1 trial sought to demonstrate the superiority of semaglutide 2.4 mg over placebo as an adjunct to lifestyle intervention (dietary counseling and increased physical activity for 150 minutes per week).^98^ This trial included 1961 participants who were randomly assigned in a 2:1 ratio to 9 semaglutide group experienced a 14.9% decrease in body weight from baseline to week 68, compared to a 2.4% decrease with placebo (*P* < .001). Additionally, 86.4% of participants in the semaglutide group lost at least 5% of their body weight, compared to 31.5% in the placebo group (*P* < .001). The STEP 3 trial included 611 participants who were randomly assigned to receive either once weekly 2.4 mg of semaglutide SC or a placebo.^[101]^ This trial sought to demonstrate the maximum effect of semaglutide by supplementing the drug with a low-calorie diet for the first 8 weeks and intensive behavioral therapy for the remaining 68 weeks. At week 68, the semaglutide group lost an average of 16.0% of their body weight, while the placebo group lost 5.7% (*P* < .001). In the semaglutide group, 86.6% of participants lost at least 5% of their body weight, compared to 47.6% in the placebo group (*P* < .001). The STEP 4 trial investigated the impact of continued treatment with 2.4 mg of subcutaneous semaglutide on the maintenance of body weight loss in addition to lifestyle changes.^[102]^ A total of 902 participants received semaglutide 2.4 for 20 weeks, with a mean weight change of 11.3 (−10.6%) kg from baseline. After the 20-week run-in period, they were randomized in a 2:1 ratio to continue treatment with subcutaneous semaglutide or placebo for the remaining 48 weeks. Participants who continued taking semaglutide after randomization lost an additional 7.9% of their bodyweight, adding up to a total weight loss of 17.4% over the course of the trial. Those who switched to placebo regained an average of 6.9%, giving a total weight loss of 5.0% (*P* < .001). The long-term STEP 5 trial lasted 2 years and involved 304 participants who were randomly assigned in a 1:1 ratio to receive semaglutide 2.4 mg or placebo, along with lifestyle interventions.^[103]^ Semaglutide significantly decreased body weight from baseline to week 104 compared to placebo (−15.2% vs −2.6%, *P* < .0001). The study found that adults with overweight or obesity were more likely to lose at least 5% of their body weight with semaglutide compared to placebo (77.1% vs 34.4%; *P* < .0001). In terms of semaglutide’s safety profile, the most commonly reported adverse effects were GI, including nausea, vomiting, diarrhea, and constipation. In all the 68-week trials, weight loss with semaglutide was accompanied by greater improvements than placebo with respect to cardiometabolic risk factors, including reductions in waist circumference, BP, C-reactive protein, glycated hemoglobin levels, and lipid levels.

#### 8.1.4. Cardiovascular outcome trials in patients with obesity

Although these increases in CV markers suggest that the use of GLP-1 RA may induce weight loss while also improving CV outcomes, trials specifically designed to investigate CV outcomes in patients with diabetes and obesity have yet to be completed. Semaglutide 2.4 mg SC once weekly, for example, is currently being investigated for the treatment of obesity in the Semaglutide Effects on Heart Disease and Stroke in Patients With Overweight or Obesity (SELECT) trial.^[104]^ This 5-year, randomized, double-blind, parallel-group trial aims to enroll 17,500 obese patients with preexisting CVD to determine whether semaglutide is superior to placebo when added to standard of care for preventing MACE. The SELECT trial is open to patients over 45 years old with a BMI of 27 kg/m^2^ or higher and a history of CVD. Patients with an HbA1c level >48 mmol/mol (6.5%), a history of type 1 or T2D, or evidence of a previous MI, stroke, hospitalization for unstable angina pectoris, or transient ischemic attack within 60 days of screening are excluded. The primary CV endpoint is the time from randomization to the first occurrence of MACE, while the confirmatory secondary endpoints are the time from randomization to CV death and all-cause death. To summarize, the SELECT trial is the first of its kind because it assesses superiority in MACE reduction for an anti-obesity medication in patients without diabetes; thus, it has a high potential for advancing an innovative approach to CVD risk reduction while concurrently combating obesity.

#### 8.1.5. Indications and dosage

In conclusion, injectable semaglutide is sold under 2 brand names: Ozempic and Wegovy. Ozempic is prescribed to adults with T2DM in conjunction with diet and exercise to improve glycemic control and reduce the risk of MACE in adults with T2D and established CVD,^[84]^ whereas Wegovy is indicated as an adjunct to a reduced calorie diet and increased physical activity for chronic weight management in adults with a BMI >30 or 27 kg/m^2^ with at least 1 weight-related comorbid condition.^[84]^ The current Ozempic guidelines recommend starting semaglutide at a dose of 0.25 mg once weekly and increasing to 0.5 mg once weekly after 4 weeks. If adequate glycemic control is not achieved after another 4 weeks, the dose can be increased to 1 mg once weekly.^[79]^ Wegovy initial dose of 0.25 mg is gradually increased over a 4-week period until a maintenance dose of 2.4 mg weekly is achieved.^[89]^ Finally, the most common side effects of these drugs included nausea, vomiting, diarrhea, stomach pain, and constipation. Oral semaglutide, Rybelsus tablets 7 or 14 mg, is prescribed once daily for the treatment of T2DM in conjunction with diet and exercise.^[81]^

### 8.2. Exenatide

#### 8.2.1. Cardiovascular outcome trials in patients with diabetes

The “Effects of Once-Weekly Exenatide on Cardiovascular Outcomes in Type 2 Diabetes” (EXSCEL) study looked at the CV effects of once-weekly exenatide in T2D patients (the median baseline HgA1c was 8%).^[105]^ This trial enrolled 14,752 patients (73.1% of whom had preexisting CVD) who were randomly assigned to receive exenatide once weekly or placebo for a median of 3.2 years. The study found that 11.4% of patients in the exenatide group experienced the primary composite outcome (3-point MACE) compared to 12.2% in the placebo group, indicating noninferiority (*P* < .001), but not superiority (*P* = .06). Furthermore, secondary outcomes (rate of death from CV causes, fatal or nonfatal MI, fatal or nonfatal stroke, and hospitalization for HF) showed no significant difference between groups. After 6 months, the exenatide group had a 0.7 percentage point lower mean glycated hemoglobin level than the placebo group. This difference narrowed over the trial period (*P* < .001). Exenatide resulted in lower mean values for body weight (−1.27 kg), systolic blood pressure (−1.57 mm Hg), low-density lipoprotein cholesterol (−1.5 mg/dL), and triglycerides (−1.8 mg/dL) compared to placebo.

#### 8.2.2. Weight loss trials in patients with diabetes

A meta-analysis of weight loss in patients with obesity and T2DM found that taking exenatide 20 mcg twice daily resulted in an additional 1.4 kg weight loss, while taking exenatide 2 mg once weekly resulted in an additional 1.6 kg weight loss when compared to placebo.^[106]^ Apovian et al^[107]^ conducted a 24-week study in which participants with obesity and T2D were given exenatide in addition to a lifestyle modification program that included a 600 kcal/d deficit and at least 2.5 hours of increased physical activity per week. In this trial, 194 patients were randomized to either 5 μg of exenatide twice daily SC with intensive lifestyle modification program or placebo with lifestyle modification program. Exenatide combined with lifestyle modification resulted in greater weight loss (−6.16 ± 0.54 vs −3.97 ± 0.52 kg, *P* = .003), hemoglobin A1c (−1.21% ± 0.09% vs − 0.73% ± 0.09%, *P* < .0001), systolic (−9.44 ± 1.40 vs − 1.97 ± 1.40 mm Hg, *P* < .001), and diastolic (−2.22 ± 1). Exenatide and lifestyle modification program participants experienced higher rates of nausea compared to the placebo and lifestyle modification program groups (44.8% vs 19.4%, respectively, *P* < .001). There was no significant difference between the 2 groups in terms of withdrawal rates (4.2% vs 5.1%, *P* = 1.0) or hypoglycemia rates.

#### 8.2.3. Weight loss trials in patients with obesity

A 35-week study of 41 obese and diabetic women was conducted to determine the effect of exenatide treatment versus placebo on weight loss.^[108]^ Results showed that subjects treated with exenatide lost an average of 2.49 ± 0.66 kg compared to a 0.43 ± 0.63 kg weight gain during placebo treatment (*P* < .01). In total, 30% of subjects responded well and lost ≥5% of their body weight (−7.96% ± 0.52%); 39% responded modestly and lost <5% of their body weight (−2.43% ± 0.45%); and 31% had no response and gained weight (1.93% ± 0.53%). The exenatide group showed greater reductions than the placebo group in systolic blood pressure (−2.48 vs −1.26 mm Hg), fasting plasma glucose (0.45 vs 3.10 mg/dL), and waist circumference (−1.68 vs 0.94 cm). Furthermore, nausea was the most common adverse effect in 56% of subjects receiving exenatide, compared to 21% of patients receiving placebo.

#### 8.2.4. Indications and dosage

In conclusion, exenatide, sold under the brand names Byetta and Bydureon, is approved as an adjunct to diet and exercise for improving glycemic control in adults with T2DM.^[78,109]^ However, Byetta and Bydureon have different dosages. For example, Byetta is injected twice daily at an initial dose of 5 mcg and is increased to 10 mcg twice daily after 1 month based on clinical response, whereas Bydureon is administered once weekly at a dose of 2 mg. Commonly reported adverse effects included nausea, diarrhea, and vomiting.

### 8.3. Liraglutide

#### 8.3.1. Cardiovascular outcome trials in patients with diabetes

##### 8.3.1.1. The liraglutide effect and action in diabetes

Evaluation of Cardiovascular Outcome Results (LEADER) trial randomly assigned 9340 diabetic participants aged more than 50 years with known CVD or over 60 years with at least 1 CV risk factor to receive liraglutide or placebo.^[110]^ The median follow-up was 3.8 years. At baseline, 81.3% of participants had established CVD, 24.7% had chronic kidney disease stage 3 or higher, and the average HgA1c was 8.7%. The primary outcome was the first occurrence of a composite CV outcome, which included death from CV causes, nonfatal MI, or nonfatal stroke. The study found that 13% of participants in the liraglutide group experienced the primary composite outcome, compared to 14.9% in the placebo group (*P* < .001 for noninferiority; *P* = .01 for superiority). The improvement in MACE was primarily due to lower CV death (*P* = .007) and all-cause mortality (*P* = .02). The liraglutide group outperformed the placebo group in terms of cardiometabolic risk factors, with 2.3 kg more weight loss, 1.2 mm Hg lower systolic blood pressure, and −0.40 percentage points lower HbA1c.

#### 8.3.2. Weight loss trials in patients with diabetes

The Satiety and Clinical Adiposity—Liraglutide Evidence (SCALE) program consists of 4 large-scale multicenter phase III trials that were conducted to determine the efficacy of liraglutide as a weight loss agent.^[111–114]^ The SCALE-diabetes trial was specifically designed to look into the efficacy of liraglutide for weight loss in obese or overweight diabetic patients.^[111]^ In this 56-week double-blind, placebo-controlled trial, 846 participants were randomized in a 2:1:1 ratio to once-daily liraglutide 3 mg, liraglutide 1.8 mg, or placebo, with a 500-kcal deficit per day and increased physical activity of ≥150 min/wk. The inclusion criteria required a BMI of 27.0 or higher, participants taking 0 to 3 oral hypoglycemic agents (metformin, thiazolidinedione, sulfonylurea) with stable body weight, and a glycated hemoglobin level of 7.0% to 10.0%. This trial had 3 co-primary endpoints: percentage change in weight, proportion of patients who lost 5% or more, and 10% or more of baseline weight. Weight loss rates were 6.0% (6.4 kg) with liraglutide 3 mg, 4.7% (5.0 kg) with liraglutide 1.8 mg, and 2.0% (2.2 kg) with placebo (*P* < .001). The proportion of patients who lost more than 5% of their initial weight was 54.3% with liraglutide 3.0 mg, 40.4% with liraglutide 1.8 mg, and 21.4% with placebo (*P* < .001). The proportion of patients who lost more than 5% of their initial weight was 54.3% with liraglutide 3.0 mg, 40.4% with liraglutide 1.8 mg, and 21.4% with placebo (*P* < .001). Similarly, liraglutide 3 (25.2%) mg and liraglutide 1.8 (15.9%) mg resulted in a higher proportion of participants losing at least 10% body weight than placebo (6.7%). Furthermore, liraglutide 3.0 mg was associated with improved glycemic control in terms of HbA1c level change, proportion of participants meeting HbA1c targets, and fasting plasma glucose level when compared to placebo. Liraglutide 3.0 mg significantly increased total cholesterol, very low-density lipoprotein cholesterol, high-density lipoprotein cholesterol, and triglyceride levels when compared to placebo. The mean systolic blood pressure was significantly lower with liraglutide (regardless of dose) than placebo. Finally, adverse events such as GI disorders (particularly nausea) and hypoglycemic episodes were more common with liraglutide 3.0 mg than liraglutide 1.8 mg or placebo.

#### 8.3.3. Weight loss trials in patients with obesity

The SCALE Obesity and Prediabetes trial was a 3-year, randomized, double-blind, placebo-controlled study. It included a 56-week initial study period followed by a 2-year follow-up on patients with prediabetes.^112^ Patients with a BMI of at least 30 or a BMI of at least 27 with treated or untreated dyslipidaemia or hypertension were considered eligible, while those with diabetes were excluded. A total of 3731 participants were randomized 2:1 to receive once-daily liraglutide 3.0 mg SC or placebo, as well as lifestyle modification counseling sessions. The co-primary end points included body weight change, proportion of participants losing at least 5%, and more than 10% weight loss from baseline. At week 56, patients in the liraglutide group lost more body weight than placebo (8.4 vs 2.8 kg, *P* < .001). Liraglutide significantly increased the proportion of patients who lost at least 5% (63.2% vs 27.1%) and 10% (33.1% vs 10.6%) of their body weight compared to placebo (*P* < .001). The fasting lipid profile improved significantly after liraglutide treatment. Liraglutide significantly decreased HbA1c, fasting plasma glucose, systolic and diastolic blood pressures compared to placebo (*P* < .001).

The SCALE maintenance trial investigated the efficacy of liraglutide in maintaining weight loss achieved with a low-calorie diet.^[113]^ Participants in this randomized, double-blind, placebo-controlled trial had a BMI ≥30 (obese) or ≥27 (overweight) kg/m^2^ with comorbidities.^[114]^ They had to lose at least 5% of their initial weight in a 4 to 12-week low-calorie diet run-in period to be considered eligible for randomization. Out of 551 participants, 422 met the goal and were randomly assigned to receive either liraglutide 3.0 mg or placebo. Throughout the trial, lifestyle interventions such as diet and exercise counseling were offered. Co-primary endpoints included the percentage change in body weight after randomization, the proportion of participants who maintained their initial weight loss of ≥5% during the run-in period, and the proportion who lost ≥5% of their randomization weight. At week 56, liraglutide significantly reduced weight compared to placebo (6.2% vs 0.2%, *P* < .0001). Patients taking liraglutide lost at least 5% of their randomization weight (50.5% vs 21.8%) and maintained a 5% run-in weight loss (81.4% vs 48.9%). Finally, improvements in some CV risk factors were observed, with liraglutide-treated participants achieving significantly greater decreases in BMI, waist circumference, mean systolic blood pressure, HbA1c, and fasting plasma glucose than patients given placebo. In the SCALE Maintenance and SCALE-obesity and prediabetes trials, the most commonly reported adverse event with liraglutide 3 mg was mild or moderate nausea.

#### 8.3.4. Indications and dosage

Liraglutide was previously marketed as an antidiabetic treatment under the brand name Victoza, but it has now been approved as a weight reduction medication under the new trade name Saxenda.^[79,87]^ Victoza is a multifunctional medication that reduces the risk of significant adverse CV events in persons with T2DM or existing CVD. Victoza is dosed initially at 0.6 mg SC once daily for a week to relieve GI problems. After 1 week, the dose is increased to 1.2 mg once daily; if glycemic control is not attained, the dose may be increased to 1.8 mg SC once daily. Saxenda is administered SC once daily at a dose of 0.6 mg, which is increased weekly until the target dose of 3 mg SC once daily is met. Both drugs have common side effects such as nausea, diarrhea, vomiting, and constipation.

## 9. Other GLP-1 RA

As for other GLP-1 RAs such as dulaglutide, albiglutide, and lixisenatide, data based on trials solely investigating the effects of these drugs on weight loss in patients with obesity is scarce; however, an abundance of trials have tested the CV effects of these drugs in patients with diabetes.

### 9.1. Dulaglutide and cardiovascular outcomes

One such trial was the Researching Cardiovascular Events with a Weekly Incretin in Diabetes trial which investigated dulaglutide’s effect on MACE in individuals with T2D.^[115]^ In this study, 9901 patients with an HbA1c >7% and proven CVD or CV risk factors, were randomized to weekly dulaglutide 1.5 mg or placebo. The primary composite outcome was the first occurrence of CV death, nonfatal MI, or nonfatal stroke. During a median follow-up of 5·4 years, the primary endpoint occurred in 12% of participants in the dulaglutide group compared with 13.4% in the placebo group, which was significant (*P* = .026). The analysis of secondary CV outcomes showed that nonfatal stroke was also significantly lower in the dulaglutide group compared with placebo (2.7% vs 3.5%, *P* = .017), however, dulaglutide did not show any effects on all-cause mortality (*P* = .067).

### 9.2. Lixisenatide and cardiovascular outcomes

The first trial which evaluated lixisenatide, published in 2016, was the Evaluation of Lixisenatide in Acute Coronary Syndrome trial which included 6068 patients with T2DM who had sustained an acute coronary event within 180 days before randomization.^[116]^ They were assigned to either lixisenatide or placebo for a median of 25 months. The primary endpoint was the first occurrence of one of the following: death from CV causes, nonfatal stroke, nonfatal MI, or hospitalization for unstable angina. Results showed an insignificant effect of lixisenatide compared with placebo on the occurrence of the primary composite outcome (13.4% vs 13.2% in the placebo group, *P* < .001 for noninferiority, p ¼ 0.81 for superiority) proving noninferiority of lixisenatide, but not superiority.

### 9.3. Albiglutide and cardiovascular outcomes

In 2018, the trial on albiglutide and CV outcomes in patients with T2DM and CV disease (Heart Attack Reduction with Medications Organized as New Therapies Outcomes) was performed on 9463 participants who were followed for a median duration of 1.6 years; all patients had a preexisting coronary, cerebrovascular, or peripheral artery disease.^[117]^ In patients receiving standard care, addition of once-weekly albiglutide reduced the risk of the primary composite outcome (3-point MACE) by 22%, compared with the addition of placebo. These results indicated that albiglutide was superior to placebo (*P* < .0001 for noninferiority; *P* = .0006 for superiority).

## 10. Clinical application of GLP-1 RAs

### 10.1. Patient selection criteria

To summarize, GLP-1 RAs are particularly effective for individuals with T2D requiring enhanced glycemic control and especially beneficial for overweight or obese patients, as they help promote weight loss.^[118]^ Approved for weight management, GLP-1 RAs, such as liraglutide and semaglutide, are recommended for individuals with a BMI of 30 kg/m² or greater or 27 kg/m² with related comorbidities like hypertension.^[119]^ They are also preferred for type 2 diabetic patients with established CVD or those at high risk, given the significant reductions in MACE seen in trials like LEADER and Semaglutide in Subjects with Type 2 Diabetes.^[97,110]^ Liraglutide also has reno-protective properties, making it suitable for patients with mild to moderate chronic kidney disease, although caution is necessary in advanced cases due to dehydration risks.^[120]^ Patient should be thoroughly screened for a history of pancreatitis or medullary thyroid carcinoma, due to GLP-1 RAs potential exacerbation of these diseases.^[121]^ When considering patient preferences, those seeking weight loss or wanting to avoid hypoglycemia risks associated with insulin or sulfonylureas are suitable candidates.^[122]^

### 10.2. Monitoring strategies

Additionally, regular monitoring plays a crucial role in maximizing treatment efficacy. HbA1c evaluations conducted every 3 to 6 months are critical for assessing glycemic control, with anticipated decreases of 0.5% to 1.5%.^[123]^ Consistent weight monitoring is also crucial, as patients may experience a 5% to 10% reduction in body weight during the initial year.^[124]^ For individuals with compromised kidney function, it is advisable to monitor serum creatinine and eGFR to avoid acute kidney injury.^[125]^ Healthcare professionals should also be vigilant for common digestive side effects, including nausea, vomiting, and diarrhea, which can be addressed through gradual dose increases.^[126]^ Although GLP-1 RAs have a low hypoglycemia risk, it is crucial to educate patients about symptom recognition, particularly if they are using other glucose-lowering medications.^[127]^ To ensure patient safety, it is essential to monitor for indications of pancreatitis and thyroid function, especially in those with a family history of thyroid cancer.^[128]^

### 10.3. Potential challenges

While GLP-1 RAs offer numerous advantages, their effective implementation in clinical settings faces several obstacles. The high cost of these medications, coupled with limited insurance coverage, can impede patients’ ability to maintain long-term treatment.^[129]^ Moreover, the need for injection may deter individuals who are averse to needles, although oral semaglutide presents a non-injectable option with specific usage instructions.^[130]^ It is important for patients to be educated on the medication and to have realistic expectations about treatment outcomes, including the timeframe for improvements in blood sugar levels and weight reduction.^[131]^ For elderly patients, it is crucial to weigh the risks associated with multiple medications and potential drug interactions, as GI side effects could increase the risk of dehydration.^[132]^

## 11. Future direction and trials

### 11.1. GLP-1 RAs in nonalcoholic fatty liver disease treatment

Concurrent with the obesity epidemic, the prevalence of nonalcoholic fatty liver disease (NAFLD) has risen dramatically, becoming the most prevalent liver disorder in Western nations.^[133]^ At present, no FDA-approved medications exist for NAFLD treatment, with the primary recommendation being lifestyle modifications aimed at weight reduction, which is often challenging to achieve and sustain.^[134]^ Consequently, innovative therapeutic advancements like GLP-1 RAs are crucial. These drugs not only facilitate weight loss but also contribute to reducing liver inflammation and fibrosis.^[135]^ For instance, liraglutide has shown promise in enhancing nonalcoholic steatohepatitis histology and decelerating fibrosis progression in the “Liraglutide Efficacy and Action in Diabetes” (LEAD) study, while semaglutide has also exhibited potential in NAFLD treatment.^[136,137]^ Despite these encouraging results, many GLP-1 RAs still require thorough investigation for NAFLD treatment, necessitating additional clinical trials to confirm their effectiveness.^[135]^

### 11.2. Emerging research areas

Another promising area of investigation is the potential neuroprotective effects of GLP-1 RAs in conditions such as Alzheimer disease. Preliminary studies indicate that GLP-1 RAs may mitigate neuroinflammation, enhance brain insulin sensitivity, and safeguard against cognitive decline.^[138]^ Further investigation into CV and renal benefits is underway, particularly concerning heart failure and chronic kidney disease, with ongoing research assessing whether these benefits extend to nondiabetic populations.^[139]^ Personalized medicine approaches are gaining traction, with research focusing on identifying biomarkers and genetic profiles to predict patient response to GLP-1 RAs, potentially improving treatment outcomes.^[140]^

### 11.3. Conclusion

The future of GLP-1 RAs is promising, with expanding indications and ongoing trials that could transform the management of metabolic, liver, neurodegenerative, and CVDs.^[133–138]^ Addressing challenges related to safety, cost, and access will be crucial to maximizing their potential in clinical practice.

## Author contributions

**Conceptualization:** Fatima Ali Raza.

**Formal analysis:** Fatima Ali Raza, Rafiya Altaf, Talha Bashir, Sohaib Tousif, Mahnoor Faisal Mohammad.

**Project administration:** Fatima Ali Raza, Sajjad Ali.

**Resources:** Fatima Ali Raza, Rabiya Altaf, Mahnoor Faisal Mohammad.

**Validation:** Fatima Ali Raza, Rabiya Altaf, Aman Goyal, Aisha Mohammed, Mahfuza Anan, Sajjad Ali.

**Writing—original draft:** Fatima Ali Raza, Rafiya Altaf, Talha Bashir, Fatima Asghar, Rabiya Altaf, Sohaib Tousif, Aman Goyal, Sajjad Ali.

**Investigation:** Rafiya Altaf, Fatima Asghar, Rabiya Altaf, Sohaib Tousif.

**Methodology:** Rafiya Altaf, Talha Bashir, Fatima Asghar, Rabiya Altaf, Sohaib Tousif, Aman Goyal, Mahnoor Faisal Mohammad, Mahfuza Anan.

**Funding acquisition:** Talha Bashir, Aman Goyal, Aisha Mohammed.

**Software:** Talha Bashir, Aisha Mohammed.

**Data curation:** Fatima Asghar, Sohaib Tousif, Mahnoor Faisal Mohammad.

**Visualization:** Fatima Asghar, Sajjad Ali.

**Supervision:** Aman Goyal, Aisha Mohammed, Mahfuza Anan, Sajjad Ali.

**Writing—review & editing:** Aman Goyal, Aisha Mohammed, Mahnoor Faisal Mohammad, Mahfuza Anan, Sajjad Ali.
